# Experimental investigation of wettability alteration, IFT reduction, and injection schemes during surfactant/smart water flooding for EOR application

**DOI:** 10.1038/s41598-023-37657-1

**Published:** 2023-07-13

**Authors:** Seyed Soheil Noorizadeh Bajgirani, Amir Hossein Saeedi Dehaghani

**Affiliations:** Petroleum Engineering Department, Faculty of Chemical Engineering, Tarbiat Modares University, Tehran, Iran

**Keywords:** Fossil fuels, Chemical engineering

## Abstract

In recent years, the application of smart water and surfactant in order to improve oil recovery has attracted special attention in carbonate reservoirs. In this research, the effects of various salts in smart water and two surfactants of Cetyl Trimethyl Ammonium Bromide (CTAB) and Sodium Dodecyl Sulfate (SDS) on the wettability alteration of carbonate rock and IFT were studied. Besides, along with micromodel flooding, core flooding tests were conducted to assess the amount of oil recovery at reservoir conditions as an injection scheme was used. In this regard, the results illustrated that the presence of CTAB or SDS in seawater (SW) can act better in contact angle reduction compared to smart water. Also, a four times increase in the concentration of SO_4_^2−^ and removing Na^+^ from SW reduced the contact angle to 68° and 71°, respectively, being the best possible options to alter the carbonate surface wettability to more water-wet states. Moreover, in the second-order process in which the rock section was first placed in SW, and then was put in the smart solution (with or without surfactant), CTAB had a great effect on the wettability alteration. In the case of IFT reduction, although SW4Mg^2+^, compared to other ions, better decreased the IFT to 17.83 mN/m, SW + SDS and SW + CTAB further declined the IFT to 0.67 and 0.33 mN/m, respectively. Concerning different ions, divalent cations (Mg^2+^ and Ca^2+^) show better results in improving oil recovery factor. However, the combination of SW and surfactants has a more positive effect on boosting oil recovery, as compared to smart water flooding. It should be mentioned that the first-order injection is better than the second-order one since SW is flooded at first, and then, after the breakthrough, smart water is injected into the micromodel. In addition, the core flooding tests showed that SW + CTAB and SW + SDS in tertiary injection increased the oil recovery to about 59 and 57%, respectively, indicating that the presence of CTAB could be more effective than that of SDS.

## Introduction

The most crucial challenge in oil production from carbonate reservoirs is that large amounts of oil, after ordinary water flooding, remain inside the rock matrix grid and adhere to the surface of the rock^1–3^. The main reason for this phenomenon is the wettability of carbonate rock, which is oil-wet, and ordinary water flooding cannot alter the wetting state of the rock surface to produce considerable amounts of oil from such reservoirs^4^. As a result, most of the oil traps inside the pores and throats of carbonate rock; thus, any enhanced oil recovery (EOR) method that can modify the initial wettability to the water-wet condition will be an effective way to increase oil recovery factor. One of the most promising novel EOR techniques to boost the oil recovery factor is smart water flooding. In fact, this cost-effective EOR method has attracted attention since not only has it less environmental issues but also it can result in more oil production^5–8^. In smart water flooding, the concentrations of ions are adjusted and optimized, and the equilibrium of the oil/brine/rock (CBR) system can be changed, thereby improving oil recovery^7–9^. There have been numerous studies in which the effect of smart water flooding on EOR has been investigated, and it has been reported that wettability alteration and calcite dissolution would be the mechanisms of ion-engineered water flooding^10–18^. Also, the interplay between divalent cations and anions has a significant impact on the CBR system. Indeed, Ca^2+^, Mg^2+^, and SO_4_^2−^ can affect the surface-active agents, including asphaltene and resin, at fluid/fluid and rock/fluid interface, leading to oil recovery enhancement^19–21^. It should also be emphasized that although smart water has a great ability to decrease contact angle, it cannot be effective in IFT reduction. Consequently, the use of surfactant in smart solutions has been suggested so as to improve oil recovery. That is to say, the addition of surfactant to smart water can further decline IFT values and mobilize the re-trapped residual oil^22–25^. Additionally, the synergy that occurs between surfactants and various ions can lead to more IFT and contact angle reductions^26,27^. As for IFT reduction, the presence of salts in the solution can heighten the solubility of oil molecules in the aqueous phase by virtue of the salting-in effect, leading to a decline in IFT values^28^. Also, surfactants at the oil/water interface form a thin film as their hydrophobic and hydrophilic parts are placed at the oil and water phases, respectively. Thus, when more surfactants get adsorbed at the interface, the film is reinforced and IFT values decline further^29^. Regarding wettability alteration, the mechanisms that are responsible for contact angle reduction could be ion exchange as surfactants are utilized^30^. Besides, in the presence of salts, salting-in effect and dissolution are the driving mechanisms to detach oil molecules from the rock surface^19,28^.

There have been some studies that explored the impact of novel smart water/surfactant flooding on EOR. In this respect, Utilizing CTAB in smart solutions with different concentrations of SO_4_^2−^, Saeedi Dehaghani et al.^31^ reported that when the concentration of SO_4_^2−^ quadrupled in brine, CTAB can act better in reducing the IFT and the contact angle. Ahmadi et al.^32^ performed contact angle tests on carbonate rock and found that 1 wt% of CTAB in brine can further decrease the contact angle compared to that of SDS and TX-100, and the combination of smart water and surfactant has a more key role in increasing the oil recovery. Also, Moradi et al.^33^ employed a natural surfactant, which was plant-based, along with potential determining ions (Mg^2+^, Ca^2+^, and SO_4_^2−^), and their results indicated that increasing the concentration of plant-based surfactant to 3 wt% (its critical micelle concentration (CMC) value) can lead to a 72% rise in the oil recovery in the presence of 4 times SO_4_^2−^ concentration. Moreover, Mofrad and Saeedi Dehaghani^34^ investigated the effects of SDS and CTAB on the contact angle when they are present in an optimized smart solution, and reported that CTAB has a big advantage over SDS in decreasing the hydrophobicity of the carbonate surface. In addition, employing cationic surfactant (DTAB) with the Mg^2+^, Karimi et al.^35^ showed that if Mg^2+^ and DTAB are available in smart solutions, a more water-wet condition can be seen. In another study, Karimi et al.^36^ observed that the synergy that occurs between SO_4_^2−^ and DTAB can alter the wettability of calcite surface to a more water-wet condition relative to the smart solution that does not have surfactant.

Therefore, as can be seen, there are limited studies in the area of smart water/ surfactant flooding to improve oil recovery, and there are still some parts that need to be addressed. This study aims to compare different smart waters with seawater containing surfactants at various CMC values so as to boost oil recovery. Besides, different injection schemes were evaluated to achieve a maximum oil recovery rate. Additionally, this study discerns how the first- and second-order processes, explained in detail later, can affect the wettability alteration. In this regard, we initially performed contact angle tests to investigate the potential of smart water, with different ions, and surfactants (cationic and anionic) in wettability alteration. Next, for each ion and surfactant, at the concentration where the contact angle was minimized, the IFT tests were conducted. Also, micromodel flooding was performed to witness wettability alteration in the pore scale and calculate the oil recovery factor when injection schemes were implemented. Finally, core flooding was done at reservoir conditions to obtain the oil recovery when a smart water/surfactant solution was injected as a tertiary recovery scheme.

## Materials and methods

### Materials

#### Fluids

The seawater (SW) utilized in this study was Persian Gulf seawater with the composition given in Table 1. By adjusting cationic and anionic ions (Na^+^, Mg^2+^, Ca^2+^, K^+^, SO_4_^2−^, and HCO_3_^−^) in SW, the desired smart water is produced. Besides, the composition of formation water is also shown in Table 1. Moreover, CTAB and SDS were employed in SW to show the effect of cationic and anionic surfactants on different aspects of enhanced oil recovery. Therefore, different Smart water solutions (with or without surfactants) were prepared to conduct varied tests, and their compositions are demonstrated in Table 2. As shown in Table 2, brines have symbols, entailing SWxNa^+^, SWxK^+^, SWxHCO_3_^−^, SWxSO_4_^2−^, SWxMg^2+^, and SWxCa^2+^. For example, SW2Na^+^ denotes that the concentration of NaCl has become doubled as compared to its initial value in SW. As for the presence of surfactants in SW, SW3SDS and SW3CTAB reveal that the surfactant concentrations are equal to their CMC values.

**Table 1:** The Persian Gulf (SW) and the formation water components.

Ions	K^+^	Na^2+^	Ca^2+^	Mg^2+^	Cl^−^	SO_4_^2−^	Sr^2+^	HCO_3_^−^
SW	399	12,000	440	1632	22,358	3110	3.3	166
Formation water	1986	42,215	5032	759	789,421	635	547	579

**Table 2:** Smart water and surfactant solutions.

Number	Solution	K^+^ (ppm)	Na^+^ (ppm)	Ca^2+^ (ppm)	Mg^2+^ (ppm)	Cl^−^ (ppm)	SO_4_^2−^ (ppm)	Sr^2+^ (ppm)	HCO_3_^−^ (ppm)	CTAB (ppm)	SDS (ppm)
1	SW	399	12,000	440	1632	22,358	3110	3.3	166	0	0
2	SW0Na^+^	399	1452	440	1632	6064	3110	3.3	166	0	0
3	SW2Na^+^	399	22,511	440	1632	38,653	3110	3.3	166	0	0
4	SW3Na^+^	399	33,059	440	1632	54,957	3110	3.3	166	0	0
5	SW4Na^+^	399	43,607	440	1632	71,241	3110	3.3	166	0	0
6	SW0K^+^	0	12,000	440	1632	22,003	3110	3.3	166	0	0
7	SW2K^+^	798	12,000	440	1632	22,713	3110	3.3	166	0	0
8	SW3K^+^	1197	12,000	440	1632	23,068	3110	3.3	166	0	0
9	SW4K^+^	1596	12,000	440	1632	23,423	3110	3.3	166	0	0
10	SW0Mg^2+^	399	12,000	440	0	17,601	3110	3.3	166	0	0
11	SW2Mg^2+^	399	12,000	440	3264	27,115	3110	3.3	166	0	0
12	SW3Mg^2+^	399	12,000	440	4896	31,872	3110	3.3	166	0	0
13	SW4Mg^2+^	399	12,000	440	6528	36,629	3110	3.3	166	0	0
14	SW0Ca^2+^	399	12,000	0	1632	21,577	3110	3.3	166	0	0
15	SW2Ca^2+^	399	12,000	880	1632	23,139	3110	3.3	166	0	0
16	SW3Ca^2+^	399	12,000	1320	1632	23,920	3110	3.3	166	0	0
17	SW4Ca^2+^	399	12,000	1760	1632	24,701	3110	3.3	166	0	0
18	SW0HCO_3_^−^	399	11,768	1760	1632	22,358	3110	3.3	0	0	0
19	SW2HCO_3_^−^	399	12,464	1760	1632	22,358	3110	3.3	332	0	0
20	SW3HCO_3_^−^	399	12,696	1760	1632	22,358	3110	3.3	498	0	0
21	SW4HCO_3_^−^	399	12,928	1760	1632	22,358	3110	3.3	664	0	0
22	SW0SO_4_^2−^	399	10,622	1760	1632	22,358	0	3.3	166	0	0
23	SW2SO_4_^2−^	399	13,379	1760	1632	22,358	6220	3.3	166	0	0
24	SW3SO_4_^2−^	399	14,758	1760	1632	22,358	9330	3.3	166	0	0
25	SW4SO_4_^2−^	399	16,137	1760	1632	22,358	12,440	3.3	166	0	0
26	SW1CTAB	399	12,000	440	1632	22,358	3110	3.3	166	100	0
27	SW2CTAB	399	12,000	440	1632	22,358	3110	3.3	166	250	0
28	SW3CTAB	399	12,000	440	1632	22,358	3110	3.3	166	370	0
29	SW4CTAB	399	12,000	440	1632	22,358	3110	3.3	166	550	0
30	SW5CTAB	399	12,000	440	1632	22,358	3110	3.3	166	700	0
31	SW6CTAB	399	12,000	440	1632	22,358	3110	3.3	166	800	0
32	SW1SDS	399	12,000	1760	1632	22,358	3110	3.3	166	0	230
33	SW2SDS	399	12,000	1760	1632	22,358	3110	3.3	166	0	1150
34	SW3SDS	399	12,000	1760	1632	22,358	3110	3.3	166	0	2300
35	SW4SDS	399	12,000	1760	1632	22,358	3110	3.3	166	0	4600
36	SW5SDS	399	12,000	1760	1632	22,358	3110	3.3	166	0	9200

It should be noted that all the materials were purchased from Merck Company.

#### Crude oil properties

The composition of provided crude oil from the Sivand oilfield is reported in Table 3. The density and viscosity of oil are 0.87 g/cm^3^ and 197 cp at 22 °C, respectively.

**Table 3:** Mole percentage of oil component.

Composition	C_2_	C_3_	iC_4_	nC_4_	iC_5_	nC_5_	C_6_	C_7_	C_8_	C_9_^+^
Mole percentage	0.01	0.03	0.78	1.83	3.99	5.95	6.82	6.22	8.21	66.16

#### Reservoir rock properties

In order to carry out the core flooding test, two different core plugs from the carbonate reservoir of Iran were used for this study, and their properties are illustrated in Table 4. Also, for measuring the contact angle, the rock was cut into thin sections with a thickness of 2 mm. The XRF analysis of the rock sample used in this research is shown in Table 5.

**Table 4:** Core properties.

Core	Length (cm)	Diameter (cm)	Pore volume (cc)	Permeability (md)
Num-1	3.8	6.4	11.38	57
Num-2	3.8	6.4	8.59	71

**Table 5:** XRF results of rock.

Material	CaO	Na_2_O	MgO	Al_2_O_3_	SiO_2_	P_2_O_5_	SO_3_	Cl	K_2_O	MnO	Fe_2_O_3_	Sr	L.O.I
Percentage	55.024	0.041	0.363	0.076	0.004	0.008	0.110	0.017	0.015	0.072	0.914	0.047	43.09

### Experimental procedure

#### Contact angle measurements

**Figure 1:**
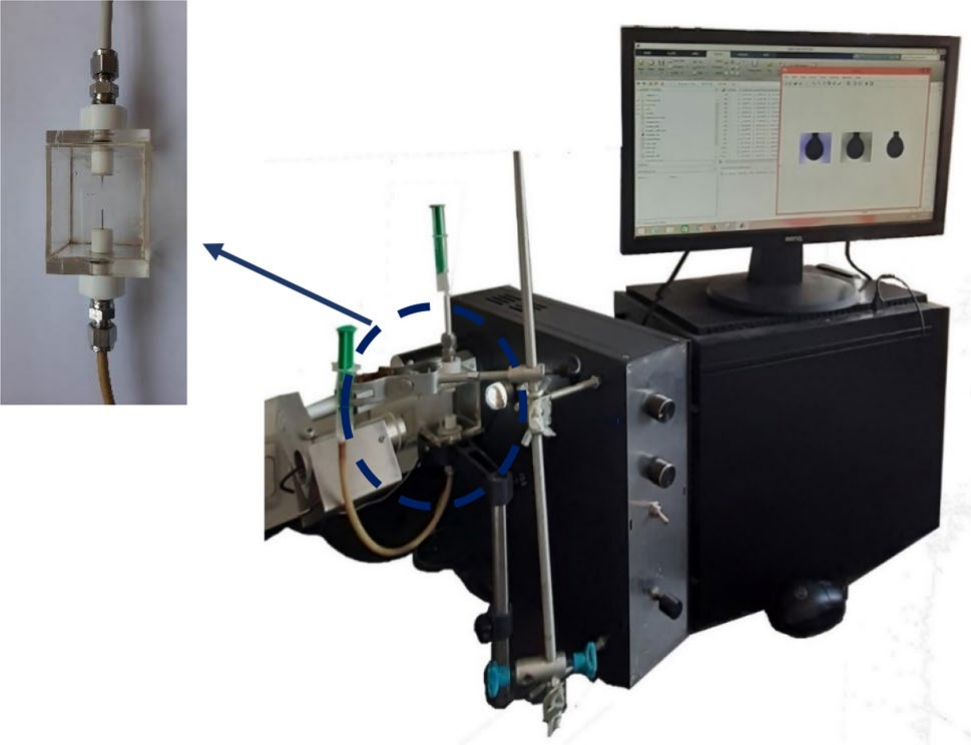
Contact angle and IFT setup.

A sessile drop technique was utilized to measure the contact angle and obtain the alteration of wetting properties of the surface. In this regard, first of all, the thin sections made from the carbonate rock were washed with toluene and methanol repeatedly so as to eliminate any excessive particles. Then, those thin sections were put in the formation water for two weeks at room temperature. Next, to make the samples oil-wet, all the thin sections were placed in crude oil for two weeks at 80 °C. Finally, each thin section was aged in desired smart solutions for 21 days, and the contact angle was measured by using a setup shown in Fig. 1.

#### Interfacial tension measurements

A pendant drop method was used to measure IFT values. In this method, a drop of oil is suspended from a curved needle, which is upward in a smart solution. After reaching the equilibrium condition, an image was taken from the suspended oil droplet, and the IFT was calculated by Eq. (1)^37^.

1 $$\sigma =\frac{\Delta \rho g{D}^{2}}{H},$$

where $\sigma$ stands for the interfacial tension, $\Delta \rho$ represents the density difference between oil and smart solution, g denotes gravity of the earth, D signifies the equatorial diameter of the oil droplet, and H is a parameter obtained based on the droplet shape. It should be mentioned the shape analysis software (LabVIEW software) was used to measure the IFT values. This method has received wide currency among researchers since it has high accuracy (0.01 mN/m)^38,39^.

#### Micromodel tests

**Figure 2:**
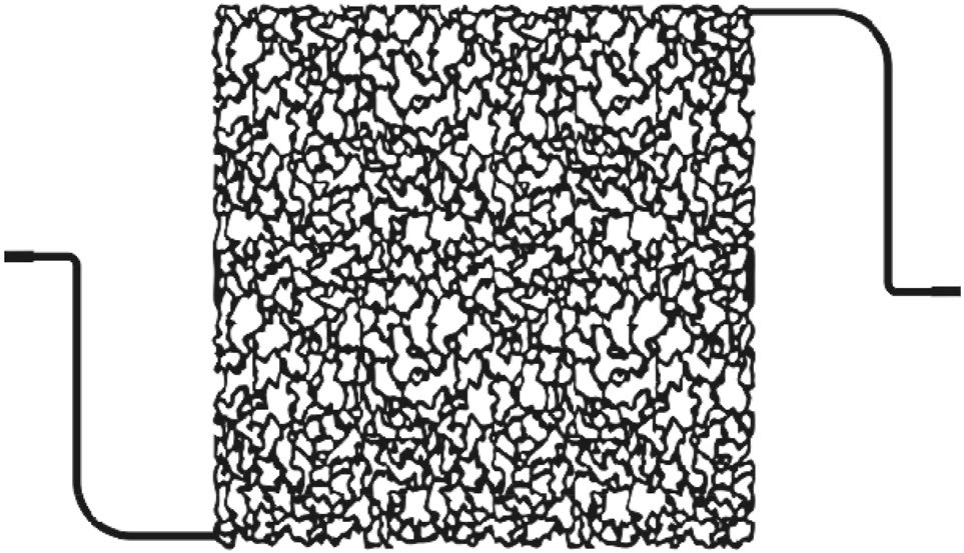
Schematic of micromodel.

In this research, a glass micromodel, which is a five-spot, was employed to calculate the amount of oil recovery and witness wettability alteration in the pore scale. The micromodel shown in Fig. 2 has a porosity of 37% and a permeability of 890 mD. In order to perform each micromodel flooding test, the model was first made oil-wet, and the following procedure was used for this purpose^34^:

1. The micromodel is saturated by a mixture containing 5% of hexamethyldisilane and 95% of toluene for 20 min.
2. The micromodel is washed with methanol multiple times.
3. The micromodel is dried at 100 °C for an hour.

After the glass micromodel became oil-wet, the oil was injected at a rate of 3 ml/h into the model to completely saturate it, and, then, the desired smart solution was flooded at a rate of 0.05 ml/h since this rate avoids turbulence behavior^40,41^. Afterward, during the flooding, the picture of the micromodel was shot by a digital camera in a specified time interval. Next, utilizing image processing (Photoshop CS6), the amount of oil recovery was calculated.

#### Coreflood tests

**Figure 3:**
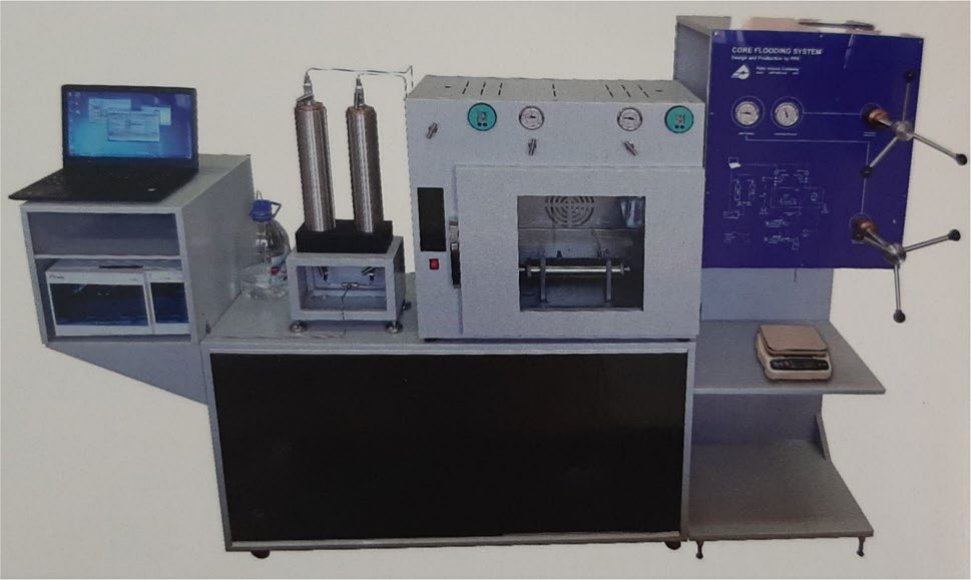
Core flooding system.

For each core flood test, after cleaning and drying procedures at 150 °C, formation water was first injected until the core was fully saturated, then crude oil was flooded. After that, as the irreducible formation water saturation was obtained, the core was aged for 72 h at 80 °C to attain an oil-wet condition. Next, each plug was successively flooded with SW, optimized smart water, and SW + surfactant at a flow rate of 6 ml/h. This test was performed at 90 °C with a back pressure of 3300 psi and confining pressure of 3000 psi. The complete core flood setup is shown in Fig. 3.

## Results and discussion

### Contact angle measurements

First of all, the effect of ions was investigated on the contact angle by changing the ions concentration in SW, and the results are presented in Table 6. For Na^+^, As can be seen, the higher the concentration of Na^+^ in the SW, the higher the contact angle will be. As the concentration of Na^+^ increased from 0 to 4 times the initial concentration, the contact angle rises by 46.2°. This is because increasing the concentration of Na^+^ leads to a decrease in the activity of divalent ions and prevents their access to the double layer, thereby increasing the contact angle^28^. Besides, the presence of Na^+^ in the double layer does not have enough ability to alter the contact angle, since it is an inactive ion. As a result, when Na^+^ is eliminated from SW, divalent ions can have easy access to the double layer, and wettability can be changed to a water-wet state due to their activity. In the case of Ca^2+^ and Mg^2+^, the addition of these ions to SW can reduce the contact angle significantly, owing to the generation of a complex structure with fatty acids. Therefore, these fatty acids are separated from the surface of the rock, and more water-wet conditions can be seen^19,38^. It should be noted that among the two mentioned divalent cations, Ca^2+^ further decreases the contact angle due to its greater tendency to approach the carbonate surface. Although the absence of Na^+^ in SW is crucial to decrease the contact angle, the optimal wettability alteration for K^+^ can be obtained as its concentration is doubled. This is due to the fact that K^+^ has greater ionic movement and lower ionic hydration; Thus, it can have better access to the surface of the rock, and the contact angle decreases through the presence of this ion^42^. However, as compared to SW0Na^+^, SW2K^+^ had less capacity to alter the wettability of the rock surface and decreased the contact angle to 79°. Moreover, quadrupling the concentration of HCO_3_^−^ in SW can reduce the contact angle to 79.2° because this ion replaces fatty acids at the surface of the rock and slightly affects the wettability, as a result of H^+^ activity. Nevertheless, HCO_3_^−^ is unable to change the wetting condition like other ions. In this study, the best reduction in the contact angle values happened as the concentration of SO_4_^2−^ was quadrupled. When the concentration of SO_4_^2−^ rises in SW, wettability changes as a result of three reasons^20^. Firstly, the surface of carbonate rock has positive charges, and SO_4_^2−^ can be adsorbed on the rock, resulting in reducing these charges. Thus, Ca^2+^ and Mg^2+^ can easily approach the surface of the rock and alter the wetting conditions. Secondly, divalent cations can form ion pairs with SO_4_^2−^ and, consequently, they are able to have easy access to the surface, as SO_4_^2−^ is adsorbed on the surface. The shapes of oil droplets in the best and worst contact angle values are shown in Table 7.

**Table 6:** Contact angle in different concentration of salts.

x	SWxNa^+^	SWxCa^2+^	SWxMg^2+^	SWxK^+^	SWxHCO_3_^−^	SWxSO_4_^2−^
0	71.1	90.9	98.5	85.9	82.0	123.6
2	91.8	91.3	88.1	79.0	88.9	79.9
3	105.1	80.2	80.8	90.3	85.3	74.3
4	117.3	75.2	75.3	92.2	79.2	67.7

**Table 7:** Shape of an oil droplet in best and worst contact angle.

	SW2K^+^	SW0Na^+^	SW4Mg^2+^	SW4Ca^2+^	SW4SO_4_^2−^	SW4HCO_3_^−^
Best angle						

	SW2K^+^	SW4Na^+^	SW0Mg^2+^	SW2Ca^2+^	SW0SO_4_^2−^	SW2HCO_3_^−^
Worst angle						

**Figure 4:**
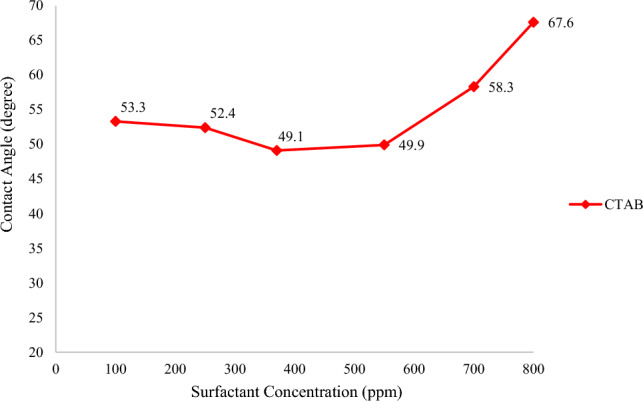
Contact angle in various concentration of CTAB.

**Figure 5:**
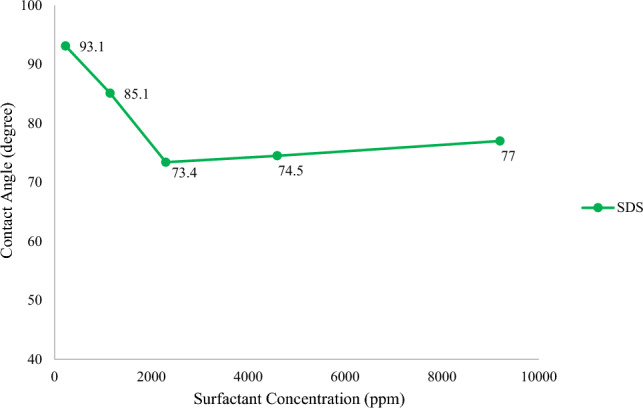
Contact angle in various concentration of SDS.

**Figure 6:**
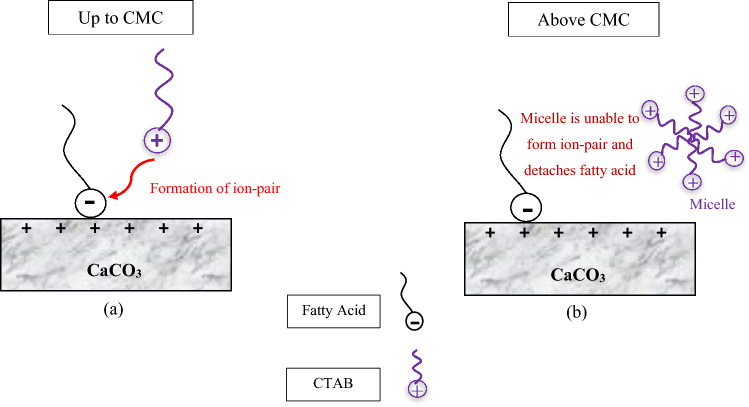
Proposed mechanism of wettability alteration by SW + CTAB: (**a**) ion-pair formation and (**b**) micelle formation.

In the next step, different concentrations of surfactants were added to SW, and the changes in the contact angle were evaluated, and the results are illustrated in Figs. 4 and 5. It should be mentioned that the CMC of SDS and CTAB was 2300 and 370 ppm, respectively^43,44^. According to Fig. 4, increasing the concentration of CTAB in SW, up to its CMC, reduces the contact angle to 49.1°. However, a further rise in the CTAB concentration, above the CMC value, contributes to an increase in the contact angle value. One of the possible reasons for this decrease and increase in the contact angle would be that CTAB has a positive charge producing a complex structure with a negative charge of fatty acid on the rock. Therefore, the fatty acids can be separated from the surface, and the wettability can be changed to a more water-wet condition. In other words, during making the carbonate surface oil-wet, the fatty acids present in the oil get adsorbed on the positive surface charge of the carbonate rock. As approaches the surface, CTAB can interact with fatty acids by electrostatic interaction, forming ion-pair^45,46^. As a result, oil molecules can be detached from the carbonate surface, and the surface becomes more water-wet. Additionally, the carbonate surface has both negatively and positively charged species. Above the CMC value, CTAB monomers tend to form micelles, and the formation of micelles hinders the positive effect of the single free monomer from altering the wettability. As a result, increasing the concentration of CTAB more than its CMC value can increase the contact angle since CTAB monomers are unable to produce ion-pairs (cationic-anionic) with fatty acids and detached them from the surface of carbonate rock. The mechanisms for the contact angle increase and decrease are presented in Fig. 6.

**Figure 7:**
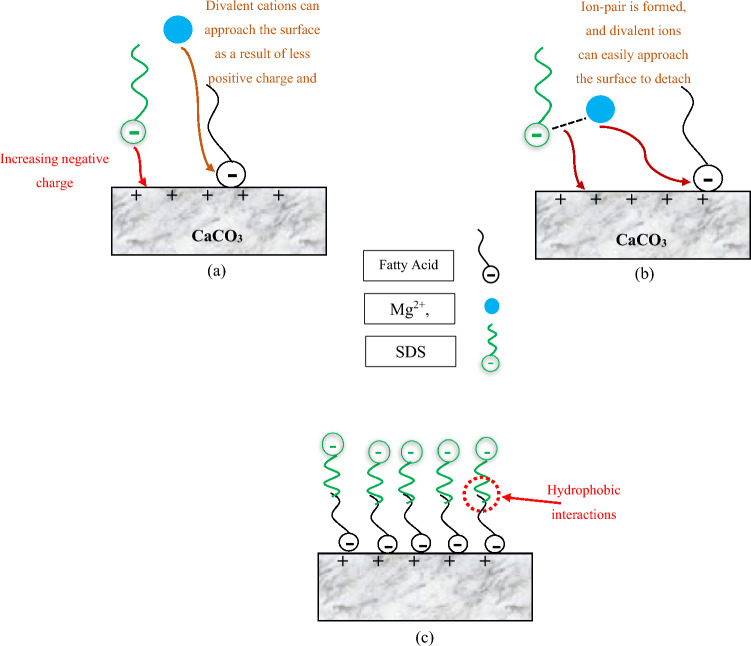
Proposed mechanism of wettability alteration by SW + SDS: (**a**) inducing more negative charge, (**b**) formation of ion-pair, and (**c**) hydrophobic interaction.

In the case of SDS, the presence of this surfactant and increasing its concentration up to its CMC value can decrease the contact angle to 73.4° (Fig. 5). This decline in the contact angle values can be attributed to three compelling reasons, as shown in Fig. 7. First of all, a monolayer is formed on the carbonate surface as tails of SDS monomers react with adsorbed fatty acids by hydrophobic interactions^47–49^. As a result, the hydrophilic parts of SDS molecules cover the rock surface, leading to more water-wet conditions. Moreover, SDS has the capacity to reduce the positive charge of carbonate rock^31,49^. To put it differently, SDS monomers have negatively charged head groups, and the surface of carbonate rock is positively charged. At the rock/brine interface, these monomers are adsorbed on the rock through electrostatic interactions and reduce the amount of positive charge on the carbonate surface. In this regard, the attractive forces between fatty acids and the carbonate surface are diminished, and negatively charged oil molecules can be detached from the surface, making it more water-wet. Also, another potential reason is that divalent cations can form ion-pair with the hydrophilic part of SDS. As a result, as SDS is adsorbed on the rock surface, more Mg^2+^ and Ca^2+^ are available in close proximity to the surface owing to the formation of ion-pairs, and the wettability can be further altered. In other words, as SDS molecules and divalent ions are present in the solution, ion-pair is created between them. SDS, owing to its intrinsic charge, can get adsorbed on the carbonate surface. Because of SDS adsorption and ion-pair formation, more amounts of Mg^2+^ and Ca^2+^ can accumulate at the rock/brine interface; therefore, fatty acids can be released due to ion exchange between divalent ions and complex structure (Ca^2+^/COO^−^). Despite this, increasing the concentration of SDS above the CMC value leads to a minimal rise in the contact angle because SDS monomers create micelles. Thus, the created micelles cannot act as single free SDS monomers to reduce the positive charge of the rock surface, and the contact angle increases due to the decreased number of divalent ions in the proximity to the surface.

It is also crucial to perceive the synergistic effects of different ions and surfactants for wettability alteration. As for CTAB and divalent anions, SO_4_^2−^ can make the rock/brine zeta potential more negative, and this facilitates the adsorption of CTAB on the carbonate rock^20^. Simply put, SO_4_^2−^ increases the negative charge of rock, and, as a result, CTAB monomers have more chance to be adsorbed on the rock surface owing to the interactions between their positively charged head groups and negative charges of the surface. Thus, the synergy that occurs between CTAB and divalent anions results in more water-wet conditions. Regarding SDS and divalent cations, Mg^2+^ and Ca^2+^ are able to render the carbonate surface charge more positive^19,28^. Therefore, it is likely that SDS molecules have more facile access to the carbonate surface due to reduced repulsive forces. Consequently, more amounts of SDS monomers can get adsorbed on the surface, and the wettability changes toward more water-wet states. Also, as stated above, ion-pair formation between divalent cations and SDS molecules could be another possible synergy for wettability alteration. Indeed, ion-pair could be formed between the positive charge of cations and the negative charge of SDS. As SDS is adsorbed on the carbonate surface, divalent cations, due to ion-pair formation, can be present at rock/brine interface and separate adsorbed fatty acids by virtue of ion exchange. Accordingly, the synergistic effect of SDS and divalent cations can lead to wettability alteration.

**Figure 8:**
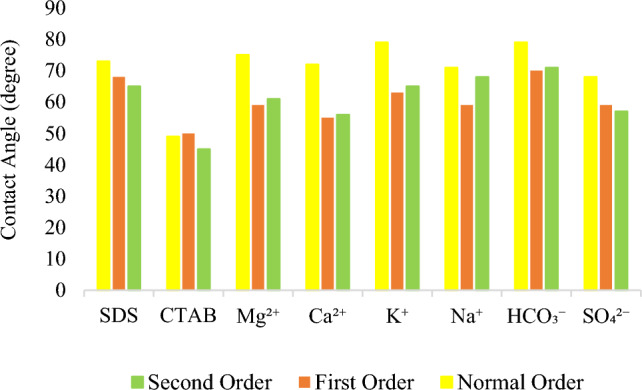
Contact angle in three different orders.

In the final step, after finding the optimum concentrations, including SW0Na^+^, SW4Ca^2+^, SW4Mg^2+^, SW2K^+^, SW4HCO_3_^−^, SW4SO_4_^2−^, SW3CTAB, and SW3SDS, the final test was performed to find out that the rock should be in contact with SW first and then with smart water or vice versa so as to reduce the contact angle further. In this regard, in the process called first-order, the small sections of rocks were placed in SW for 4 days, and, next, they were put in the smart solutions with optimum concentrations for another 4 days. Then, the contact angle was measured by the sessile drop technique. Besides, at the same time, other small rock sections were first put in the smart solutions, and then they were placed in SW. This reverse process is called second-order. The results are presented in Table 8 and Fig. 8. As can be seen from Fig. 8, in most cases, the contact angle decreased due to the longer permanence of the rock in the solutions. In this way, a longer reaction happens between the rock and fluid, and thus its effect on wettability is greater. Accordingly, the first-order and second-order can be better in reducing contact angle as compared to previous tests where the rock fragments were just positioned in one solution.

**Table 8:** Contact angle in dfferent orders.

Smart solutions	SW3SDS	SDS3CTAB	SW0Na^+^	SW4Ca^2+^	SW4Mg^2+^	SW2K^+^	SW4HCO_3_^−^	SW4SO_4_^2−^
The first-order	68	50	59	55	59	63	70	59
The second-order	64	45	68	56	61	65	71	57

As shown in Table 8, with regard to Na^+^, the first-order is more effective in altering the wettability than the second-order. In fact, in the first-order, SW first reduced the contact angle. Subsequently, the activity of divalent ions was increased by placing the rock in the smart solution, which didn’t have Na^+^, and the contact angle decreased further. However, in the second-order, in which the rock was first put in the smart solution (SW0Na^+^), the contact angle value declined, but in the next step, when it was placed in SW, wettability could not be changed significantly. In respect of divalent ions (Ca^2+^, Mg^2+^, and SO_4_^2−^) and other monovalent ions (K^+^ and HCO_3_^−^), the contact angle was almost equal whether the process was the first-order or the second-order.

In the case of surfactants, the second-order has a more positive impact on reducing the contact angle. This is because, in the first-order, as the rock section is placed in SW, the contact angle decreases, and some ions are adsorbed on the rock, owing to their activity. In the next step, when it is put in the smart solution, surfactants are unable to approach the rock surface and further decline the contact angle, as a result of adsorbed ions. Therefore, if the first-order is utilized, the presence of surfactants in SW can be less effective in decreasing the contact angle values, as compared to the second-order.

### Interfacial tension tests

**Figure 9:**
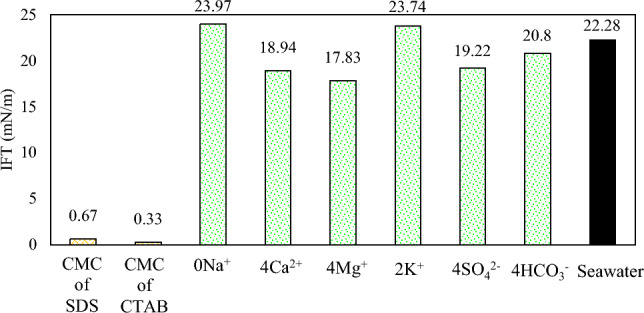
IFT in optimum smart solutions.

**Figure 10:**
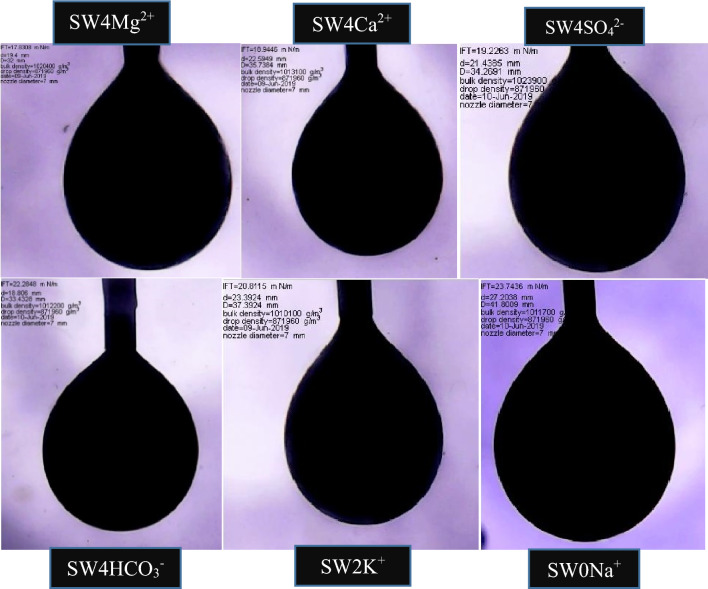
Oil droplet shapes during IFT measurements between optimal smart solutions.

**Figure 11:**
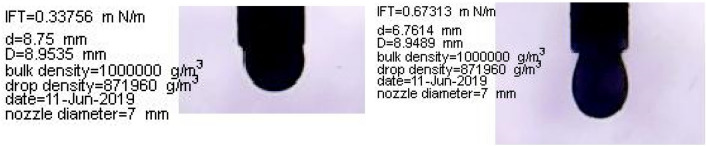
Oil droplet shapes during IFT measurement by CTAB (left) and SDS (right) solutions.

IFT tests were conducted at the optimal concentrations, obtained from the contact angle tests, and the results are shown in Fig. 9. According to Fig. 9, the combination of SW and surfactants can contribute to the lowest IFT values. The IFT value in SW3CTAB and SW3SDS was 0.33 and 0.67 mN/m, respectively. Therefore, it can be inferred that CTAB can play a more crucial role in reducing IFT, in comparison with SDS. The reason for this better performance is that CTAB can form complex structures with fatty acids at the oil/water interface, owing to its positive charge. Also, CTAB monomers tend to migrate from the aqueous phase in order to place at the interface. As a result, the IFT decreases alongside the presence of different ions in SW. However, complex structures cannot be formed between SDS and fatty acids since both have a negative charge. Thus, the IFT reduction can be attributed to the activity of ions and the positioning of SDS at the oil/water interface. In the case of different ions, it is clear that the absence of Na^+^ and doubling the concentration of K^+^ increase IFT values relative to SW. Therefore, the results illustrate that the presence and absence of monovalent ions cannot have positive impacts on IFT reduction since they are not determining ions due to their low activity. Quadrupling the concentration of Ca^2+^ and Mg^2+^ decreases the IFT by 3.34 and 4.45 mN/m, respectively. Therefore, it can be inferred that Mg^2+^ can act better in the IFT reduction, as compared to Ca^2+^. The reason for this condition could be that the activity of Mg^2+^ is higher, due to its smaller size and higher positive surface charge. Consequently, This ion, by entering into the stern layer and reacting with fatty acid groups and the aggregation of these groups at the interface, would further reduce IFT values. Besides, in comparison with HCO_3_^−^, more reduction in IFT values can be seen when the concentration of SO_4_^2−^ in SW is quadrupled. This is because SO_4_^2−^ is a potential determining ion that has a great ion activity; thus, it has more ability to reduce the IFT. The shapes of suspended oil droplets are shown in Figs. 10 and 11.

### Flooding tests

In this section, micromodel tests have been performed to obtain the oil recovery factor, and the results are presented in Table 9. As SW was injected into the micromodel, oil recovery increased to 19%. According to Table 9, concerning different ions, divalent cations (Mg^2+^ and Ca^2+^) show better results in improving oil recovery factor. In this respect, SW4Ca^2+^ and SW4Mg^2+^ can increase oil recovery by 19 and 20%, respectively. The reason for this is that Ca^2+^ and Mg^2+^ not only can decrease the contact angle to around 75° but also can reduce IFT values better than other monovalent and divalent ions. It should be noted that although SW4SO_4_^2−^ had the lowest contact angle value, it was unable to decrease the IFT more than did SW4Ca^2+^ and SW4Mg^2+^ solutions. Therefore, increasing the concentrations of Mg^2+^ and Ca^2+^ acts better in enhancing the oil recovery factor. Besides, among monovalent ions, the absence of Na^+^ rose oil recovery by 6%, as a result of the highest IFT and contact angle values. Moreover, the combination of SW and surfactants has a more positive effect on boosting oil recovery, as compared to smart water flooding. In other words, the presence of SDS and CTAB in SW can increase oil recovery to 55 and 70%, respectively. As can be seen from the results, if CTAB is present in SW, the highest oil recovery factor can be obtained, since it can contribute to the lowest IFT and contact angle values.

**Table 9:** The amount of oil recovery by micromodel flooding.

Solution	SW + CTAB	SW + SDS	SW 0Na^+^	SW 4Ca^2+^	SW 4Mg^2+^	SW 2 K^+^	SW 4HCO_3_^−^	SW 4SO_4_^2−^
Recovery (%)	70	55	25	38	39	30	32	35

**Figure 12:**
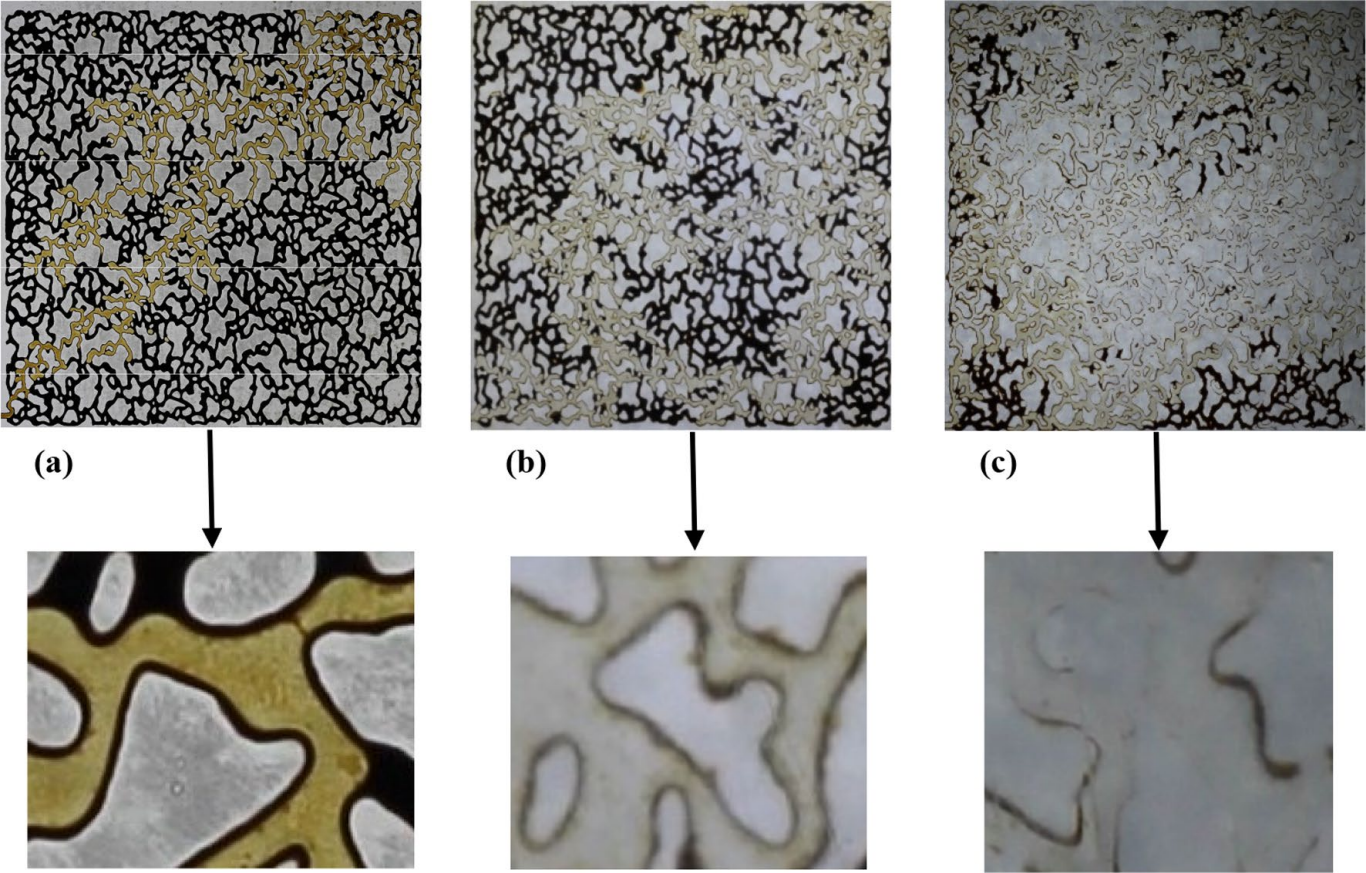
Macroscopic and microscopic images of micromodel flooding: (**a**) SW flooding, (**b**) SW4Mg^2+^ flooding, and (**c**) SW + CTAB flooding.

Figure 12 presents the image of micromodels after SW, SW4Mg^2+^, and SW + CTAB were flooded in the micromodels. According to Fig. 12a, due to SW flooding, most of the oil continues to exist in the pores of micromodel, and the fingering phenomenon happens. However, as shown in Fig. 12b, quadrupling the concentration of Mg^2+^ in SW can boost sweep efficiency. Besides, it has more capability to overcome capillary pressure since SW4Mg^2+^ has the least IFT and can change the wettability to the water-wet condition. Consequently, more oil is produced, and the fingering phenomenon is reduced as well. It should be noted that, in the microscopic image of Fig. 12b, it can be seen that the thickness of oil on the wall of pores is reduced, indicating that SW4Mg^2+^ can shift the wettability from oil-wet to water-wet. Moreover, based on Fig. 12c, it can be inferred that there is a substantial rise in oil production, as a result of SW + CTAB flooding. To put it differently, the presence of CTAB in SW can enhance sweep efficiency more than does SW4Mg^2+^, and less amount of oil remains in the micromodel. In fact, since SW + CTAB has the lowest IFT and contact angle values, it can easily penetrate pores and overcome capillary pressure, resulting in more oil production and less fingering phenomenon. Also, as can be seen from the microscopic image of Fig. 12c, there is no oil on the wall of pores and throats, illustrating that the wettability of micromodel is completely changed to water-wet condition.

Afterward, injection schemes have been performed in two ways. Firstly, SW was injected, and then smart water was flooded, which is called a first-order injection. Secondly, a second-order injection has been conducted in which the process was the opposite of the previous one. The results of oil recovery after the first-order and second-order injections are presented in Table 10. In the first-order injection, after the breakthrough of SW, smart water is injected in the second stage. As shown in Table 10, the combination of SW and CTAB resulted in the highest oil recovery factor, which is 32%. Besides, if SW + SDS was injected in the micromodel after SW flooding, the oil recovery can be increased by 19%. Therefore, it can be concluded that the presence of surfactants in SW can boost the oil recovery factor in the second stage of flooding. Moreover, increasing the concentration of divalent ions in SW can increase oil recovery from 8 to 10%, and SW4Ca^2+^ flooding in the second stage is more effective. However, in the case of monovalent ions, the absence and presence of Na^+^, K^+^, and HCO_3_^−^ in the second stage of flooding have not great impact on increasing oil recovery, and the amount of oil recovery has not changed significantly. In the second-order injection, as SW is injected in the second stage after the breakthrough of smart water, there is no substantial rise in the amount of oil recovery, and the changes are from 1 to 5% for all the smart solutions. To put it differently, SW tends to select a path that has already been swept by smart water. Accordingly, in the second stage, most of the oil will remain in the micromodel, and SW is unable to produce more untouched oil.

**Table 10:** The amount of oil recovery in secondary injection in the first-order and second-order.

Ions	CTAB	SDS	HCO_3_^−^	SO_4_^2−^	Na^+^	Ca^2+^	Mg^2+^	K^+^
Oil recovery (%) in secondary injection (the first-order)	32	19	4	9	3	10	8	4
Oil recovery (%) in secondary injection (the second-order)	3	2	5	1	1	3	4	3

**Figure 13:**
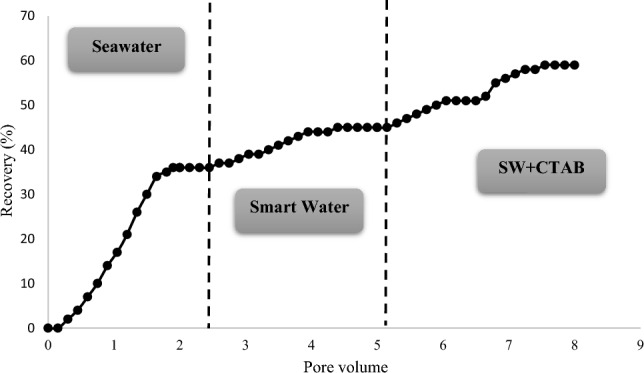
Oil recovery in three different stages of core flooding (core Num-1).

**Figure 14:**
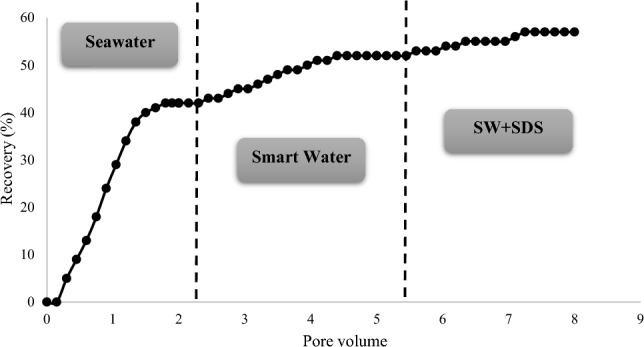
Oil recovery in three different stages of core flooding (core Num-2).

In addition, core flooding was performed to obtain the oil recovery factor when optimum solutions were injected, and the results are shown in Table 11, Figs. 13 and 14. In this respect, each core sample was first flooded with SW (2 pore volumes). Next, 3 pore volumes of SW4Mg^2+^ and SW + surfactant (CTAB or SDS) solution were injected respectively. Figure 13 presents the oil recovery when the fluids are injected into core Num-1. As can be seen, 2 pore volumes of SW increased oil recovery to 36%. Afterward, optimum smart water (SW4Mg^2+^) was flooded, and an oil recovery of 9% was obtained. Finally, after the injection of SW + CTAB, an incremental oil recovery of 14% was achieved. Also, the same test was conducted with core Num-2, and, instead of CTAB, SDS was employed to show the effect of the anionic surfactant on the oil recovery from carbonate rock. The result of this test is shown in Fig. 14. Thus, according to Fig. 14, there was an increase in the oil recovery to 42%, as a result of SW flooding. Then, in the second stage of flooding when SW4Mg^2+^ was injected, the oil recovery was increased by 10%. Eventually, SW + SDS flooding resulted in an incremental oil recovery of 5%. In both SW and smart water flooding, Core Num-2 achieved a better oil recovery factor. The reason for this could be that core Num-2 had better permeability, and, consequently, SW and optimum smart water flooding in the first and second stages of injection produced more oil from core Num-2. Despite this, in tertiary injection, SW + CTAB flooding has a more positive impact on the oil recovery in core Num-1, as compared to the injection of SW + SDS in core Num-2. Therefore, it can be inferred that cationic surfactant has a better effect than anionic surfactant on producing oil from carbonate rock. This is because the presence of CTAB in SW can change the wettability toward more water-wet conditions, and reduce the IFT value more than does that of SDS.

**Table 11:** The results of coreflooding experiments.

Core No	Injected fluids	Oil recovery after primary injection (%)	Oil recovery after secondary injection (%)	Oil recovery after tertiary injection (%)
Num-1	Seawater Smart water SW + CTAB	36	9	14
Num-2	Seawater Smart water SW + SDS	42	10	5

## Conclusion

In this study, the effect of SW, smart water, and SW + surfactant on wettability alteration, IFT, and oil recovery was examined. According to the reported results, in the case of smart water, quadrupling the concentration of SO_4_^2−^ and eliminating Na^+^ can be more effective in contact angle reduction. Besides, the presence of CTAB in SW has the greatest impact on wettability alteration relative to the smart solution and SW + SDS. It should be mentioned that when the surfactant is present in SW, the second-order process in which the rock fragments are placed in SW + surfactant, and then they are positioned in SW contributes to a more water-wet condition. Moreover, compared to monovalent ions, increasing the concentration of divalent ions further reduces the IFT values. Additionally, the presence of SDS and CTAB in SW can further decrease the IFT values as opposed to smart solutions. Among different ions, a more increase in the oil recovery can be seen if the concentration of Ca^2+^ and Mg^2+^ is quadrupled. In comparison with smart water flooding, SW + CTAB can increase oil recovery to 70%. In tertiary injection, SW + CTAB and SW + SDS increased the amount of oil recovery to 14% and 5%, respectively; Therefore, it can be concluded that cationic surfactant might be a better option to boost the oil recovery than anionic surfactant in carbonate reservoirs.

## Data Availability

All data generated or analysed during this study are included in this article. Email for contact: asaeedi@modares.ac.ir.
